# Biomarker-guided antifungal stewardship policies for patients with invasive candidiasis

**DOI:** 10.18502/cmm.4.4.385

**Published:** 2018-12

**Authors:** Behnam Honarvar, Kamran Bagheri Lankarani, Mehdi Taghavi, Ghasem Vahedi, Esmaeil Mortaz

**Affiliations:** 1 Health Policy Research Center, Institute of Health, Shiraz University of Medical Sciences, Shiraz, Iran; 2 Mycology Research Center, Faculty of Veterinary Medicine, University of Tehran, Tehran, Iran; 3 Department of Immunology, Faculty of Medicine, Shahid Beheshti University of Medical Sciences, Tehran, Iran; 4 Division of Pharmacology, Utrecht Institute for Pharmaceutical Sciences, Faculty of Sciences, Utrecht University, Utrecht, the Netherlands

**Keywords:** Antifungal agents, Antimicrobial stewardship, Biomarkers, Candidiasis, Invasive fungal infections

## Abstract

Invasive fungal infections (IFIs) are among the life-threatening issues in patients with impaired immune system. High administration of antifungals in these patients imposes a heavy economic burden on the national health system. In addition, despite the usually expensive antifungal regimens, the mortality rate due to fungal infections is still high, resulting in the loss of hundreds of lives per year.

Survival rate is an indicator of the success of national healthcare policies. Early diagnosis of IFI is critical because any delays may be fatal. The weakness of the old-fashioned culture-based diagnostic methods lies in their time-consuming laboratory procedures. To overcome this problem, several diagnostic approaches have been developed to facilitate the early diagnosis of invasive candidiasis as the most prevalent IFI.

These methods are based on the detection of serologic and molecular footprints. However, nowadays, antibiotic resistance and proper and cost-effective use of antibiotics are given special attention in national healthcare policies. The instructions for controlling these indices have been collected under the name of antibiotic stewardship. The present review study was targeted toward providing insight into novel diagnostic biomarkers and antifungal stewardship programs. The simultaneous investigation of these two issues facilitates the achievement of a novel health policy for the treatment of systemic candidiasis in immunocompromised patients.

## Introduction

Systemic candidiasis has been recently raised as one of the most prevalent nosocomial infections in immunocompromised patients [1]. *Candida* species have been known as the fourth microbial cause of nosocomial infections and the third most frequent cause of nosocomial bloodstream infections in the Intensive Care Units (ICUs) in epidemiologic studies. 

*Candida albicans* is responsible for life-threatening infections in most of the patients; however, other *Candida* species have been also increased alarmingly in recent years [2]. Systemic candidiasis is definitely a lethal disease, particularly among immunocompromised patients, resulting in a substantial mortality rate of 40-60% [3]. 

The prognosis of this infection is closely associated with early diagnosis and adherence to a proper therapeutic regimen [2, 4-7]. Clinical investigations have shown that the adoption of a timely and proper therapeutic approach facilitates the achievement of an increased survival rate and better therapeutic outcomes [8, 9].

Since clinical manifestations of systemic candidiasis are not disease-specific, it is difficult to make a proper diagnosis only based on clinical examination [3]. The old-fashioned traditional culture-based methods have remained as the only accepted gold standard for infection diagnosis. However, modern mycology can support the old methods by the aid of some state of the art and ultra-sensitive techniques, such as the molecular and immunoserological detection methods. 

The general approach to achieve a proper diagnosis of invasive candidiasis (IC) is mainly based on blood cultures. Although the overall positive results of the test reportedly account for 50% of total candidiasis cases [10-12], the blood culture is sensitive enough to detect the presence of any viable *Candida* species in the blood.

However, it becomes a matter of controversy when the blood sample is collected from a patient with deep-seated candidiasis with no or low evidence of viable *Candida* species in the blood [11]. In these cases, the blood culture fails to detect the infection [11]; therefore, there is a critical requirement for using additional diagnostic methods, along with blood cultures. 

Recent studies have introduced new findings, including interesting diagnostic biomarkers and novel disease management and monitoring strategies [13-15]. These tests mainly concentrate on serological techniques for the detection of fungal cell wall components (e.g., mannan, galactomannan and [1,3]-β-D-glucan [BG]). Molecular detection of microbial genetic remnants is another promising and successful diagnostic method for the rapid detection of *Candida* infections [16-19].

Therefore, timely diagnosis of IC greatly helps reach a better therapeutic outcome and enhanced survival rate [8, 9]. On the other hand, wrong diagnosis, false results, or overestimation of infection intensity might lead to the overconsumption of antifungal agents or improper selection of drugs [20]. This eventually increases the chance of developing resistant fungal strains and leads to a dramatic raise in healthcare costs [20, 21]. 

A variety of solutions have been suggested in response to these negative aspects of antibiotic-based therapeutic strategies. One candidate which has rendered promising results and is successfully employed in clinical settings is known as antibiotic stewardship program (ASP) [22]. With this background in mind, the present review study was conducted to discuss the new diagnostic biomarkers, their most significant features (e.g., sensitivity and specificity), and their application in the development of novel antifungal stewardship programs (AFSPs) against systemic candidiasis without the risk of increasing the chance of developing resistant strains and raising healthcare costs. 

Although much effort has been made by the researchers to achieve early diagnosis with the aid of novel biomarkers, there are few, if any, reports about the use of biomarkers as the basis for ASP. In the current study, our goal was to hypothesize and introduce a novel biomarker-guided ASP for hospitalized patients with IC.

***(1,3)-β-D-Glucan***

The BG is the key structural component of fungal cell wall in most of the fungi, including *Candida* species. Nevertheless, some fungal species, such as *Zygomycota* and *Cryptococcus* species, have a lower BG content in their cell walls. *Candida* BG remnants, released into the circulation, can be readily detected in patients’ serum during IC. The BG detection assay has been developed in the same manner as that of the limulus test [23]. The method is mainly relied on the colorimetric detection of coagulation reaction, following the activation of factor G, a protease zymogen of limulus amebocyte lysate [24]. 

Regarding the reliability and clinical significance, BG test has been mentioned as a diagnostic modality for the detection of *Candida* infections in the European Organization for Research and Treatment of Cancer/Mycoses Study Group diagnostic criteria [25]. In a meta-analysis, the pooled sensitivity and specificity of BG assays, retrieved from various studies reporting patients with proven IFIs, were estimated as 79.1% and 87.7%, respectively. Nonetheless, the pooled sensitivity and specificity of the patients with proven probable IFIs were reported as 76.8% and 85.3%, respectively, in a number of other studies [24]. 

Furthermore, it has been demonstrated that BG assays have a much higher sensitivity than the conventional blood or tissue culture [11, 12, 26, 27]. However, it should be considered that each test method has a different cutoff value (20-80 pg/ml). These variations are mostly related to the degree of the affinity of each assay to BG [28-30]. In addition, false positives might be seen when using the samples related to blood or blood products, immunoglobulin infusions, hemodialysis, surgery, ß-lactam antibiotics, and infection with *Pseudomonas aeruginosa* [31]. 

Although BG is known as a panfungal diagnostic marker, it suffers from some methodological weaknesses and false positive results. The structural similarity between the glucans of bacterial organisms and *Candida* species and the presence of bacterial remnants in circulation are responsible for the achievement of high false positive results [32]. In addition, the reliability of BG-based method for monitoring the treatment response is not fully characterized yet; however, some studies have considered this criterion. 

For example, in a study conducted on 52 patients with candidiasis, the BG levels were decreased in antifungal-treated patients [33]. However, BG is recommended as a reliable combinatory diagnostic method for *Candida* species, along with culture-based evaluation tests [11, 31, 34].

***Mannan***

Mannan as a type of polysaccharide is another major part of *Candida* cell wall. In this regard, they form about 7% of *Candida* cell wall [35]. Mannans, like BG, show antigenic potentials; therefore, they could be used for the serologic diagnosis of IC [11, 36]. Mannans are composed of mannopyranose units, and their oligomannose sequences correspond to epitopes specific for the antibodies of both human and animal sources [3]. 

Use of mannan antigens, which are usually present in the blood, during infection to develop an immunoassay for the diagnosis of systemic candidiasis was first suggested about four decades ago [37]. Mannan detection in the blood is an enzyme-based immunoassay. A commercial kit has been developed based on mannan antigens. 

Although mannan detection tests have shown a high specificity, the detection of mannan antigens is limited due to their rapid clearance from bloodstream. This problem affects the sensitivity of the mannan tests [11, 38]. Moreover, a combination assay, composed of mannan antigens and anti-mannan antibody, fails to overcome the sensitivity problem because most of the patients with IC also suffer from immunodeficiency, and therefore cannot produce enough antibodies against mannan antigens [31, 38].

***Candida DNA***

*Candida* DNA-based detection method is found to be a potentially powerful and highly sensitive diagnostic approach [11, 18]. Several molecular techniques, focusing on the detection of *Candida* DNA in the blood and tissues, have been evaluated so far [11, 18, 39, 40]. For instance, Neguyan et al. suggested a sensitivity of 89% for the in-house PCR detection of deep candidiasis [41]. 

There are two commercially available PCR tests for candidiasis, namely SeptiFast and fully automated multiplex T2*Candida* panels. However, these tests have not been fully accepted and require more validation and standardization procedures to be established as reliable diagnostic tools [11, 42, 43]. In addition, a highly sensitive and specific molecular diagnostic technique, called peptide nucleic acid (PNA) fluorescent in situ hybridization, has been developed in recent years [44]. 

The technique is based on the rapid identification of specific microbial rRNA in less than an hour through the in-situ binding of a fluorescent-labeled PNA, followed by a microscopic investigation of the fluorescent signal of hybridized PNA/microbial nucleic acid target [44]. In this technique, there is no requirement for the amplification of the target nucleic acids. The PNAs show a high affinity for binding to their complementary nucleic acid targets. This binding is even stronger than that of the usual DNA/RNA probes [44]. 

This technique shows a superior sensitivity (up to 99%), specificity (up to 98%), and positive predictive value (99%) in case of the identification of ICs with different clinically important *Candida* species [45]. Despite having many advantages, this method also entails some weak points. This technique cannot discriminate between colonization and infection unless it is designed specifically to detect the targets that are only released during the infection. Furthermore, the investigation of slides/blood smears through PNA fluorescent in situ hybridization requires a fluorescent microscope, which is usually expensive [44].

***Anti-mannan antibodies***

Identification of antibodies against *Candida* mannan is another choice among serodiagnostic tools with a good accuracy. There is a commercial enzyme-linked immunosorbent assay kit, named Platelia (manufactured by Bio-Rad Laboratories), which is based on the detection of mannan antibody. The assay can detect the circulating anti-mannan antibodies with a high specificity, but rather low-moderate sensitivity (40-70%) [11, 45]. 

Continuous release of mannan from the antibody-antigen complexes and the temporary period of mannanaemia (i.e., the rapid clearance of mannan from circulation) are the main factors affecting the sensitivity of the test [11, 45]. In addition, a low production of antibodies in immunocompromised patients, who are under immunosuppressing treatments, and inability to distinguish between the fresh and past infections are the other weaknesses of this technique [11, 45].

Regarding this, this test is suggested to be combined with at least another diagnostic method/tool (e.g., mannan antigen, BG, and *C. albicans* germ-tube antibody [CAGTA]) given its insufficient sensitivity. The diagnostic strategies combining mannan-antibody detection with another method can achieve a good overall accuracy (both sensitivity and specificity) in comparison to the single-biomarker diagnostic methods [46, 47].

***Candida albicans germ tube antibody***

The CAGTA is another currently available *Candida* species detection technique (along with anti-mannan antibody) that is based on antibody investigation. The idea behind the technique is the detection of antibodies against several hyphae-specific antigens (e.g., Hwp1) on *C. albicans* germ tube (hyphal form) [48]. The test functions properly in the detection of all *Candida* species, including non-*C. albicans* species; however, it has a lower sensitivity in the detection of non-*C. albicans* species [11, 45]. 

In most of the cases, the specificity of the method is very high. Moreover, the sensitivity of this technique reaches to 100% when the infection is a deep-seated candidiasis due to *C. albicans *[49]. A prominent advantage of CAGTA method is its capacity to discriminate between the superficial colonization and infection [45].

The weakness (i.e., low sensitivity for non-*C. albicans*) of this method can be covered by its application in combination with other serologic tests, such a BG assay [49, 50]. There is a commercial product (VIRCLIA® IgG MONOTEST, Vircell, Spain) for measuring the serum CAGTA level. This product works based on indirect chemiluminescent immunoassay to evaluate the IgG against a hyphal antigen. 

***Antimicrobial stewardship programs***

The discovery of antibiotics was a huge milestone in medicine history, which saved countless lives. However, nowadays, we are faced with some of the negative aspects of antibiotic chemotherapies, such as antibiotic resistance, emergence of microbial strains with intrinsic or acquired resistance, and heavy expenditures, resulting from the overuse or wrong selection of antibiotics [51, 52]. 

In addition, recent substantial advances in our medical knowledge have opened new survival opportunities to patients suffering from diseases considered as incurable by physicians in the past 20 years [53]. Introduction of new medical techniques and drugs provided new survival chances for the immunosuppressed (e.g., patients with HIV and neutropenia) and ICU patients, as well as those with cancer who receive immunosuppressive chemotherapies and the patients subjected to heavy surgeries, organ or hematopoietic stem cell transplantations, and central venous line/catheter or total parenteral nutrition [54]. 

Moreover, the new formulations of old antifungals, such as the one developed in liposomal amphotericin, and introduction of highly effective and completely novel class of antifungals (e.g., echinocandins) are good examples of our remarkable progress in medical mycology [55, 56]. However, from another point of view, the enhancement of survival rate and quality of life have been obtained at the expensive price of the emergence of new resistant fungal strains and imposition of a heavy economic burden on the national healthcare system [21, 57]. 

These issues led the clinicians and researchers to develop a variety of new formal solutions in the healthcare institutions and hospitals with a central goal of optimizing the antibiotic use [52]. The instructions targeted toward the establishment of antibiotic controlling strategies in healthcare settings were named ASP [58]. 

First attempts to improve the antibiotic usage were established at Hartford Hospital in late 1970s and 1980s by the participation of an infectious disease physician and a clinical pharmacist as the core members of prospective audit and feedback ASP [59]. This team introduced novel strategic healthcare concepts, such as transition therapy and streamlining (now called antibiotic de-escalation) [59]. 

Next studies, such as a randomized trial, showed that the use of antibiotics could be significantly reduced without any considerable negative effects on the success of antibiotic regimen and patient’s health [59]. In 2007, the Infectious Diseases Society of America and Society for Healthcare Epidemiology of America introduced official guidelines and instructions to establish a successful, yet customizable, ASP in the healthcare settings [59]. 

Later, many of the national organizations and authorities, as well as the World Health Organization, also confirmed and advised the ASP guidelines as a necessary healthcare policy to be established in hospitals and healthcare institutions [59]. Two ASP strategies have been developed over time by different research groups. In ASP, prospective audit with intervention and feedback (also known as back-end strategy) refers to applying some modifications in the therapeutic procedures in terms of the antimicrobial drug selection, dose, route, and duration after the initiation of an antimicrobial treatment [59]. The second ASP strategy is pre-authorization ASP (also termed as front-end strategy) denoting granting the accessibility to the selected antimicrobial after the evaluation of its appropriateness [59].

## Antifungal stewardship policy structure

To reach an optimal ASP, it is required to establish a medical group comprised of three medical specialists in each healthcare institution. The specialists needed in the team include a clinical pharmacologist with a professional doctorate degree in pharmacy and fellowship experiences in infectious diseases (in case of IFI, a fellowship in fungal diseases), a board-certified infectious disease physician, and a doctorate-level clinical microbiology specialist (in case of IFI, with a level degree of experience in mycological laboratory procedures) [58, 60, 61]. 

However, to establish a successful ASP, multifaceted methods should be adopted in a healthcare institution. The core members of ASP are preferred to be full-time employees in the institution in which the ASP is implemented [58]. A clinical mycology laboratory specialist (CMLS) plays a critical role in a biomarker-guided ASFP [22].

Early diagnosis of systemic candidiasis with the aid of novel, sensitive biomarkers helps other members of the team to properly design the following procedures of ASFP targeted toward the optimization of antifungal use and prevention of antifungal resistance emergence without the loss of the desired clinical outcome [7, 62, 63]. Use of a preauthorization-based ASFP strategy (i.e., front-end strategy) facilitates the CMLS to direct the whole ASFP team towards a successful clinical outcome and an optimized antifungal therapy by the proper selection of a biomarker with a high sensitivity and specificity [62].

***Problems of conventional methods of identification and treatment of invasive candidiasis***

A definitely high crude mortality rate of 46-75% has been reported in patients with candidemia by several retrospective studies [20]. The high mortality rate implies candidemia as a lethal disease [20]. Hospitalization and antifungal treatment have been estimated to result in an overall cost of $40,000 per case [64]. The healthcare costs attributed to *Candida* infections range from $6,214 to $142,394 [62]. Prompt identification of IC can lead to a higher chance of survival and right selection and adequate use of antifungals [62]. 

It was reported that the adequate use of antifungals in patients with IC lowers mortality rate by 33% [7]. A retrospective study showed that each 1-hour delay in antimicrobial treatment reduces the survival rate by 7.6% per hour [7]. Accordingly, the huge impact of delays as small as 12 h on survival rate has been indicated in several studies [6, 7, 9, 22]. In this regard, a retrospective cohort study showed a 3-fold increase in mortality rate after a 12-hour delay in the initiation of antifungal therapy in patients with bloodstream infection of *Candida* [6]. 

The gold standard for the diagnosis of IC is blood culture; however, this method lacks an appropriate sensitivity [10]. The sensitivity for blood culture for the detection of IC has been estimated as 50-76% [7, 11]. In addition, this method suffers from its time-consuming procedures. In this respect, this method requires at least 24 h of incubation to present the results. Some certain species, such as *C. glabrata,* need even more time [65, 66]. 

The accepted treatment strategy against IC based on guidelines is currently systemic antifungal therapy (SAT), usually with echinocandins or fluconazole [67]. In addition, empirical or prophylactic SAT was recommended by guidelines immediately after the emergence of clinical symptoms attributable to IC (i.e., suspected IC cases) [6, 68, 69]. However, these therapy regimens cost a lot [56, 70, 71]; in this regard, anidulafungin and caspofungin cost $112 per 50 mg vial and 395$ per 70 mg vial, respectively. Furthermore, the cost of one-day intravenous administration of fluconazole has been estimated as $88 [70]. 

Moreover, late diagnosis prolongs the length of hospital stay, thereby increasing the expenditures of services related to room, board, nursing, laboratory, and facilities [62]. Moreover, the only way to save patient’s life in case of late diagnosis and in the absence of an ASFP is the adoption of an expensive empirical or prophylactic SAT [60, 72]. 

According to a number of studies, a higher delay in the initiation of antifungal therapy in patients with *Candida* bloodstream infection is accompanied with a higher mortality rate [6, 9]. The mortality rate among the patients receiving antifungal therapy 12 h after a positive blood culture was only 11.1%; however, this rate underwent a three-fold increase after a 52-h delay [6]. 

***Benefits of biomarker-guided antifungal stewardship policies***

Serologic tests based on the detection of *Candida* biomarkers, such as mannan, BG, and nucleic acid assays, are superior to blood culture method in terms of both sensitivity and specificity [11, 12]. In addition, early detection is another advantage of the application of serologic/nucleic acid methods [6, 9, 27]. Use of mannan methods reportedly showed positive results for candidemia in 73% of the samples at least 2 days before obtaining positive blood cultures [73]. 

With the aid of BG assays, the ASFP team can construct preemptive therapies instead of general empirical therapies in neutropenic patients with refractory fever [68, 74-76]. These patients are predisposed to develop IFIs, such as invasive aspergillosis or IC [74]. In a study, the results of intention-to-treat and evaluable-episode analyses showed that preemptive treatment strategy in neutropenic patients, receiving broad-spectrum antibiotics, saved 11% and 14% of the patients, respectively, from empirical antifungal therapy. This means a huge reduction in treatment costs and lower chance of developing antifungal resistance [75].

Preemptive strategy reduced 35% of the expenditures related to antifungal treatments in neutropenic patients [74]. It was found that a well-designed and successful ASFP could cause a substantial reduction in antifungal use and therapeutic expenditures through shortening echinocandin treatment by 2 days and saving $1,013 per patient [77]. In this regard, an ASP in a healthcare institution facilitates saving $2,251,976 as antifungals cost in a 3-year period [78]. 

The biomarker-guided preemptive therapy of IFI (e.g., IC) seems to be a promising approach having such benefits as the optimized use of antifungals, lower chance of antifungal resistance development, lower healthcare costs, shorter length of hospital stay, and higher survival rate [63].

## Conclusion

The IC has become one of the major causes of morbidity and mortality in immunodeficient patients in recent years [79]. The most significant measures in the treatment of invasive fungal infections, especially IC, include early diagnosis and monitoring response to antifungal therapy [7]. Late diagnosis can lead to the overuse of antifungals, emergence of antifungal resistance due to infection with fungal strains prone to resistance, and even mortality. 

Since culture methods are very time-consuming, new non-culture and rapid diagnostic methods should be used. On the other hand, even a timely diagnosis by means of novel biomarkers may be accompanied with the over-administration of antifungals or prolonged/continuous use of antifungals despite the clearance of infections. This would result in the emergence of resistant strains due to the inappropriate selection of antifungal agents. 

The search for the identification of an effective solution for these problems has resulted in the introduction of ASFPs. The ASFP can be a combinatory therapeutic strategy, along with the methods facilitating the early diagnosis of IFIs, particularly the ICs. This policy has the advantages of reducing the costs of antifungal treatment, decreasing the acquired antifungal resistance, and improving the survival rate. 

According to clinical studies and sensitivity and specificity evaluations, BG assay is the most reliable method for the detection of systemic candidiasis. The BG assay can act as a part of an ASP for the treatment of systemic candidiasis in immunodeficient patients. The superiorities of this novel method make it eligible to be implemented as an important technique in the national health policies. 
